# You can’t just bring people here and then not feed them: A case in support of Indigenous-led training environments

**DOI:** 10.1177/26349825221133096

**Published:** 2022-11-17

**Authors:** Vanessa Ambtman-Smith, Koral Wysocki, Victoria Bomberry, Veronica Reitmeier, Elana Nightingale

**Affiliations:** Western University, Canada

**Keywords:** Indigenous health, training environments, decolonizing, relational, Indigenous health geographies

## Abstract

By and large, academic research in geography has advanced the colonial project, and been synonymous with extractive and reductionist research practices that subjugate Indigenous people. To counteract these harmful impacts and produce research that supports the needs of communities, advancing Indigenous sovereignty over research is vital. By presenting a case study of an Indigenous research space at a Canadian University, we argue that Indigenous training environments are more than a shared, physical space; they provide essential emotive and relational spaces of collaborative learning, wherein trainees practice relationship-building, reciprocity, and accountability. This article argues that decolonizing academic spaces dedicated to Indigenous geographic research will be essential to meeting the ethical imperative of Indigenous control over knowledge production. There is a current deficit of culturally appropriate spaces that support both the whole person and their learning. We highlight the impact of Indigenous training environments in nurturing respectful, long-standing relationships with peers, community, and research partners; a critical element of Indigenous geographies, yet one of the most challenging aspects of upholding meaningful and decolonizing research. By drawing on our diverse perspectives and research projects, we reflect on how an Indigenous-led training environment, rooted in Indigenous ways of knowing, can contribute to relational accountability both within and outside of these spaces. As more communities assert their authority over these processes, the need for respectful research grows, and it is anticipated that this article will provide a useful guide and support for emerging Indigenous training environments.

The things that I really think are important about bringing people together, offering space for people to come in and do the things that they have to do and be together—it’s critical. And if you can share food and laugh about stuff and have a place of belonging, those are really important things. (Chantelle Richmond, *8 February* 2022)

## Introduction

The buzz to write this article started in the fall of 2021, at a regular meeting of the Indigenous Health Lab (Lab), where meeting face-to-face, albeit socially distanced, had come to be an exciting and rare occurrence as we navigated through the restrictions with each wave of COVID-19. Because we were meeting in person, lunch had been ordered, and we had arranged our chairs to be 2 meters apart, centered around our meeting table, with a laptop to zoom-in students who lived remotely. It was common to have shared food or drink when we met in person, as it served to nourish our physical bodies, as much as the gathering together served to nourish us socially, emotionally, mentally, and spiritually. In relation to the title of this article, a cultural understanding of bringing people together “in a good way” has roots across many worldviews and cultures and in this article, it serves as a metaphor for the Lab itself, and how the levels of support go beyond meeting academic goals, and mirror those needs that must be nourished to create this type of specialized training environment. These gatherings also helped to break through the isolation of graduate studies, and the stress of balancing coursework, TA-ship, thesis work, and research requirements. At our meeting, the energy was high, and despite our physical distance from one another, and the barrier created through our masks, we rejoiced at the fact that we were together once again, after what had felt like an eternity of isolation. Our graduate experiences over the past 2 years had been relegated to working remotely, from our homes, and meeting virtually through digital platforms. At this meeting, we heard there was to be a Special Issue in the geography publication *Environment and Planning F*, and that the focus was to be on Indigenous Research Sovereignty, centering relational approaches to research! This was exciting news because it felt so aligned with the work of the Lab, and as we started to talk about ideas for articles, the Lab’s Director started to write down ideas on the white board. There were ideas coming from individual scholars related to their research efforts, and then there were a couple of ideas for collaborative pieces—including this piece, which grew out of a collective desire to document our experiences in the Lab. We felt inspired to write this piece because we felt grateful to be part of this Lab, and noticed a sizable gap in literature around Indigenous health training environments, at least from the perspectives of graduate students. From this initial discussion, five trainees from the Lab came together to present a case study of an Indigenous-led training environment at a Canadian University.

Our reflections center the collective view that Indigenous training environments are more than a shared, physical space; these environments center Indigenous worldviews, Ways of Knowing and Doing, values, and priorities in both the work taking place and in building capacity for trainees within the academy. In this article, we describe how the Lab is an essential emotive and relational space of collaborative learning, wherein trainees practice relationship-building, reciprocity, and responsibility for decolonizing research practices required to meet the ethical imperative of Indigenous research sovereignty over knowledge production. Through our individual and shared experiences, we highlight the impact of Indigenous training environments in nurturing respectful, long-standing relationships with peers, community, and research partners.

### Who we are

In the geographies of Indigenous health, it has become common practice to describes oneself and their relationship to the research they are undertaking as one’s positionality. From a research perspective, there is often mention of one’s epistemology, ontology, and axiology, as they impact chosen methods and influence the research relationships themselves. We believe it is critical to position oneself as it authenticates the perspectives drawn on in authoring this article, and also articulates *why* we must position ourselves. To position oneself is to enact a relational accountability, to oneself, to community, to the Ancestors before us, and to future generations who will come after us. Reflecting on positionality is a cognition of one’s human, environmental and spiritual relationships, values, ethics, history, culture, biases, relationships, strengths, weaknesses, and so on, and is not static; by taking the time to locate ourselves, we invite you, as the reader, to enter into a respectful relationship with us (Kovach, 2021; Wilson, 2008).

As five co-authors, hereafter referred to as “we,” the content and perspectives for this publication are both personal and practical. Though we come with diverse and varied life experiences, we have come to co-locate ourselves within the Lab. Collectively connected through decolonial approaches, our research projects span across varied, spatially-oriented Indigenous health geographies, connected through concepts of Indigenous health, the environment and well-being, emphasizing Indigenous community self-determination. In meeting weekly and bi-weekly for 3 months, we self-identified our own roles and responsibilities in this writing process. The first author self-identifies as an Indigenous woman (she/her) of mixed Nehiyaw–Métis and European ancestry, an Indigenous adoptee and Sixties Scoop survivor raised in large, urban areas. She has reclaimed identity through teachings, education, ceremony, and learning from Elders. As a former Indigenous health leader, she draws from her own observations and experiences within healthcare systems. The first author is in her final year of training as a PhD Candidate.

The second author is a settler woman (she/her) of Hungarian, Ukrainian, English, and Scottish ancestry. She is grounded within her roles as a daughter, granddaughter, sister, fiancé, and aunt. Her deep love for the environment, experience working in the field of environmental restoration, and education in regenerative ways of organizing have drawn her to pursue a graduate doctoral degree in Geography and Environment under the supervision of Dr. Chantelle Richmond. She is in her first year of the PhD program.

The third author (she/her) is Kanyen’kehá:ka—Yakohskaré:wake né:ne Ohswekén’:en (Mohawk—Bear Clan from Six Nations of the Grand River). She is a second-year master’s student and one of the newer members to the Lab. She describes her positionality as rooted in Haudenosaunee worldview and teachings. Her scholarship is shaped through growing up on reserve and witnessing the myriad of social injustices experienced and persevered by her nation. Her role in this article has been in contributing reflections, and as an editor.

The fourth author is a settler of Polish ancestry (she/her), and is a daughter, sister, sister-in-law, niece, dog-mom, and aunt. She is a second-year MA student who describes herself as naturally curious, open minded, and willing to learn. She has contributed as a writer, reviewer, transcript editor, and figure developer. The fifth author (she/her) is a Jewish settler scholar in the final year of her PhD program. As a new mother, her role in this publication has been to support the team as a sounding board throughout the process.

In co-authoring this piece, we share our perspectives on the Lab as a way of honoring our gratitude in belonging to this unique training environment; we believe it has propelled significant personal and professional growth in us all, and has enabled us to engage in respectful research and relationships. The reflections shared by the co-authors have been voluntary and are not meant to be inclusive of all voices within the Indigenous Health Lab (IHL), past and present. Rather, this article offers a snapshot of current experiences, observations, and questions on how a training environment helps to shape us as critical, Indigenous and allied scholars within the discipline of geography. We would like to recognize the immense and generous support and leadership of Dr. Chantelle Richmond, IHL Director, and Katie Big-Canoe, IHL Coordinator, who make the Lab the supportive environment that it is.

### Situating the IHL: Room 3107, a room for the “whole-self”

At first glance, the IHL may look similar to other academic workspaces—dedicated desks for graduate students, a computer, printer, and various office supplies. However, once inside the Lab, you are welcomed by the comforting fragrance of Wiingashk (Sweetgrass—one of four sacred medicines), photos from students’ community-based research projects and retreats, and a multitude of Indigenous art. The placement of a large table in the center of the room invites collaboration, creativity, and conversation. Lab trainees and mentor gather around this table for weekly meetings to discuss individual and shared research work but also to share meals and crafting sessions, often inviting peers from beyond our research team to take part in these social activities. The Lab is an intentional training environment nourishing the social, mental, emotional, spiritual, and cultural needs of our students.

The mission of the Lab was derived from a desire to cultivate and contribute to Indigenous knowledge production in the areas of Indigenous health, well-being and the environment. Over time, the values of Lab have evolved through experience in knowing “how to do the work” respectfully and responsibly, honoring community self-determination, practicing reciprocity, and drawing on traditional teachings and Anishinabe principles of being a good relative. As a research lab and training environment, the purpose of the Lab is not only to fulfill the social need for belonging, it also bring Indigenous critical scholarship methodologies into focus, propelling students to learn deeply about the geographies of Indigenous health, with a deliberate focus on how to conduct Indigenous health research “in community-centered approaches [and] led by ethically responsive methods” (Richmond, 2016: 153).

## Methodology

**Figure 1.**
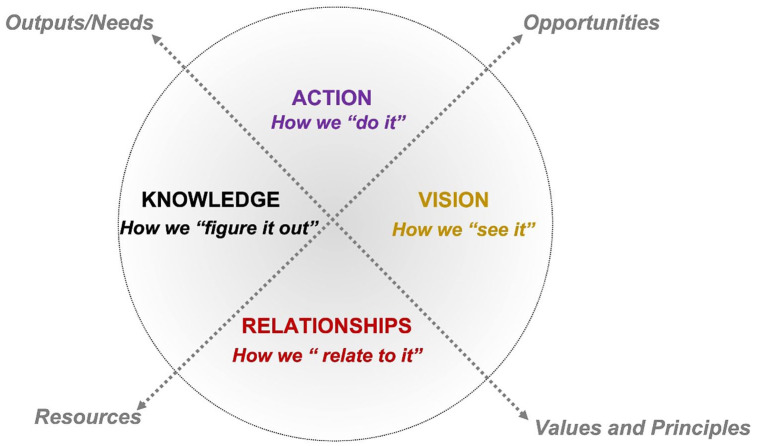
Vision Wheel process.

It is through the application of this Vision Wheel approach (see Figure 1), that we have been able to draw out connections of our individual and collective roles, responsibilities, and values enshrined in our “reality” as trainees and scholars within the Lab context. Given the focus on Indigenous research sovereignty, it is necessary to center Indigenous voice in this article; therefore, we note the lead author of this article is an Indigenous scholar, and it is through this location and lens that the methodology was derived, building on her identification as a Cree–Métis woman, who has adapted teachings through her cultural lens. The Vision Wheel exemplifies these teachings, building on the wholistic, interconnected facets of the Medicine Wheel (Elder Mary Lee, Nehiyawak) (Lee, 2012).

This article privileges Indigenous Ways of Knowing through the relational practices and values in how knowing, learning, and research is referenced. Explicitly, an Indigenous framework (the Vision Wheel) and Indigenous methods (storytelling and a sharing circle) were used to draw out and organize the reflections and storytelling from the students’ perspectives, and in establishing the flow and format for this article.

The four main sections comprising the body of this article are organized based on the architecture of the four quadrants of the Vision Wheel: Vision—how we “see it;” Relationship—how we “relate to it;” Knowledge—how we “figure it out;” and Action—how we “do it.” While we have found a sense of unity in voice, we also recognize the value and need to distinguish among the Indigenous and allied scholars’ voices in this article and have noted this where applicable.

**Figure 2.**
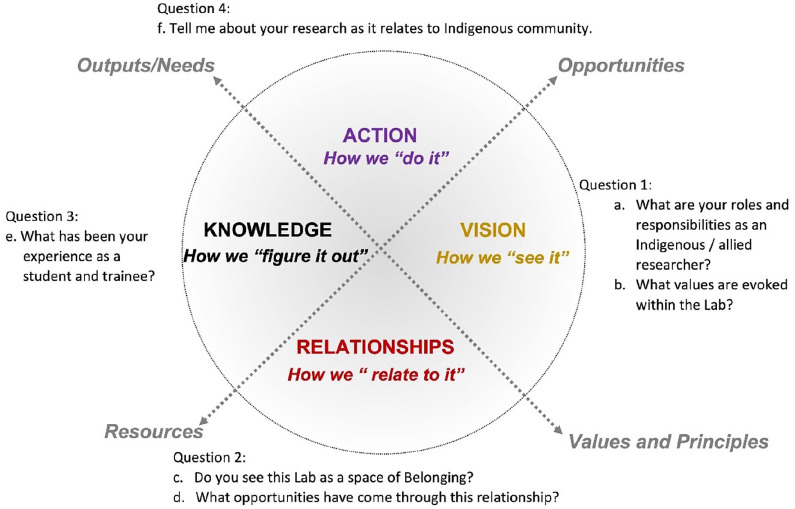
Vision Wheel process including guiding questions of the Sharing Circle.

In applying the Vision Wheel to frame the article, we are honoring the context in which knowledge was shared during the Sharing Circle, serving to support the order and content of the questions (see Figure 2). Sharing Circles are classified as a qualitative method as they do not require the collection of numerical data and are often related to the Western concept of focus groups. However, Sharing Circles differ from the focus group approach as the process goes beyond gaining knowledge through discussion and relates to the relationships that are formed during this process of ceremony (Graham and Martin, 2016; Hunt and Young, 2021). In this mode of inquiry, participants are engaged in “the gathering of stories, exploring lived experiences and in existential phenomenological inquiry or narrative research, which consists of a range of methods, including ethno-biography, analyzing biographies, and narrative interviewing” (Lavallée, 2009: 28). A Sharing Circle involves situating the group itself in a circle, even virtually, and carries sacred significance within many Indigenous worldviews (Archibald, 2008; Wilson, 2008).

In addition, within this circle, the knowledges that surface are done so using a “healing method” that draws from a spiritual base, and it is through this process of connection that the facilitator is given permission to report on the discussions. The concept of maintaining unity of information, regardless of placement, is central to this methodology, with the inherent desire to demonstrate a connected derivation of knowledge without hierarchy. There is an important rationale for employing an Indigenous conceptual framework for research as it upholds an Indigenous positioning in the research itself, beyond the specific methodologies.

Our reflections are shared following the design and movement of Figure 1, starting with the first quadrant “Vision,” located in the eastern direction. Based on our methodology, we have organized the body of the article in keeping with the following sections, as depicted through the Vision Wheel:

1. Our “Vision” of how we view our roles and responsibilities as Indigenous and allied scholars located within the IHL and how this relates to our purpose in advancing Indigenous research sovereignty efforts;
  - The origins of the Indigenous Health Lab;
  - The values and principles we have learned and practice through the Lab and how this influences our research relationships both within and outside of the training environment;
  - Learning and practicing decolonizing, geographic health research: why this matters.
2. The “Relationships” that we have cultivated through the Lab, and how these relationships serve to support relational approaches and relational accountability;
  - How we navigate through colonial tensions.
3. We share the “Knowledge” of how we “figure it out” by offering examples from our own research projects, centering Indigenous and community knowledge and reflecting on how we have come to practice research “in a good way.”
  - Examples of Knowledge—our research;
  - Enacting Indigenous knowledges and worldviews;
  - Opportunities and Challenges.
4. In the Discussion, we spotlight key learnings, questions, and recommend future opportunities for “Action”—how we “do it,” resulting from our own analysis of these tensions and opportunities.

As students, we recognize the challenges inherent in collaborating, being vulnerable, and sharing about our own journeys and experiences with the Lab, while in the midst of this journey. We believe our ability to share and work positively together was strengthened through this Indigenous, relational framework. Relational work is complex and brings out both opportunities resulting from “unity of environment,” as well as tensions associated with carrying out decolonizing work within colonial environments.

## The origins of the Indigenous Health Lab

In discussion with Dr. Richmond, we realized the roots of the Lab extend much deeper than its physical manifestation; this space was purposeful, rooted in principles of relationality and designed with a desire to redress the shortcomings experienced in their own pathway to becoming an Indigenous health scholar. Access to appropriate spaces was a central theme throughout Dr. Richmond’s training, where they recognized how fulfilling it was to have space that was Indigenous-led, nurtured belonging and celebrated Indigenous identity. Though social and cultural connections were important, an additional rigor in training was needed to redress struggles encountered “when attempting to bridge these powerful practices within the wider university context, where the same openness to Indigenized ways of learning and doing has not been similarly embraced” (Richmond, 2020).

## Vision: How we “see it”

Indigenous Ways of Knowing and Doing are central to the mission of the IHL and mirror the relational values so crucial in meeting the moral and ethical imperatives of Indigenous sovereignty in research. Drawing from the Vision Wheel framework and asserting both our individual views on how we “see ourselves” (as Indigenous and allied researchers and scholars) within the Lab, we present our narrative of what we are calling “Vision:” why we do what we do, what problem it addresses. Specific views on individual roles and responsibilities between the Indigenous and allied scholars across this vision are defined here.

### All scholars’ vision

1. Be critical scholars and “good contributors” through respectful research: all authors’ articulated the goal to enhance Indigenous research sovereignty by examining the role of decolonizing research environments that will prepare us to do research ‘in a good way’, meaning that we can attend to community needs in ways that are helpful, not harmful.
2. Interrogation as a relational practice: Russell-Mundine (2012) argues that to support a decolonizing approach to research, reflexivity is not enough; we must also intentionally interrogate Western colonial systems, environments, and practices. There is a duality in this—the work you do on the “inside” must also be done, and related to, the work on the “outside.” In our sharing circle we discussed at length our values, which have been shaped by our cultures, relationships, and past experiences. We also agreed that outside of our lab space, in the greater contexts of health geography and colonial institutions, even the idea of having values is not always welcomed, reflecting the hierarchal, paternalistic, empirical and extractive roots of contemporary Western geography (Livingstone, 1992).

### Indigenous scholars’ vision

3. Indigenize institutional practices and space:

In the lab space . . . it does create a level of safety that allows me to enact my own full role and responsibility, which is to contribute towards decolonizing the discipline (of geography) and looking at reworking the research process as a whole, so that it honors and centers the communities who are being impacted by the decisions, the structures, the environments . . . through colonial society. It will never take away from the fact that I, as an Indigenous person, as a researcher, have a lot of value to offer because I am coming from a place of knowledge and learning that has been cultivated throughout my whole life. And I think it’s so critical, to be located in a place [Lab] that values that experience, and that knowledge, and also recognizes that I don’t need to try to create a “separation” from “who I am” and, and “how I do this work”; it’s all valid. (*Author 1, Sharing Circle*)

### Allied scholars’ vision

4. Support and Advocacy: To support advancements of community knowledge and well-being; this commitment has also been described as “being held accountable” to being a responsible researcher who will “advance community knowledge and agency.”
5. Challenge Mindsets and Education: One allied scholar described the need to “use privilege to advocate from the inside-out.” An example of this advocacy is related to “using voice to speak out about harms of colonial violence,” and “seeking to be a good ally through ongoing efforts aligned with decolonizing academic infrastructure,” amplifying Indigenous voice, and educating/challenging mindsets when appropriate.

These goals align with a purposeful vision—meeting and attending to community needs through self-determination and seeking to support helpful and hopeful research. Articulating “Values” is important as we seek to critically examine our own motives and positionality (Ball and Janyst, 2008). This reflexivity is a precursor in building ethical research relationships and enacting our moral imperative as responsible and respectful scholars. Within the Sharing Circle, students were asked to “describe what values were evoked within the Lab”; here is a sample of how these values have been articulated:

### Scholars’ shared values

1. Relational: Central in the discussion was many descriptions of and examples of the value of “relationality,” which, while described more within a scholarly context in the next section of this article, through the sharing circle was described in specific terms and connected to the notions of “belonging,” “cultural safety,” “unity of environment,” “connections to others,” “love/emotion,” “patience,” “community,” and “listening.” Relationality, as described by one participant:

requires more regrowth and more learning and more relationships . . . so, I guess those are the values . . . it’s a lot of reflecting, it’s a lot of hard work, it’s a lot of learning how to communicate and being patient too, and a lot of listening (Author 4, Sharing Circle).

### Indigenous scholars’ values

2. Responsible:

My commitment is first and foremost to my community. I know that anything that I do I’ll be held accountable back at [my home community]. And so, a lot of that drives the work that I do and it drives who I am in the Academy as well. So, when I hear something that is offensive or puts my community down, I feel a responsibility to speak up. Or puts down Indigenous Knowledges as a whole, I feel like I can’t sit in a conversation that erases [us] or doesn’t acknowledge the ability and the truth in our knowledges. (*Author 3, Sharing Circle*)

3. Gratitude and Cultural Safety:

I can see very honestly that I came into this place not feeling safe and being very concerned that there wouldn’t be a place for me, and I am really grateful and honored to be surrounded by people like you all, who I learn from everyday. And . . . we have a mentor and a leader that works their butt off to make it [LAB] available for us, because without her, the space wouldn’t be here and I wouldn’t have come to [university]. (*Author 1, Sharing Circle*)

4. Protective:

So, I feel a bit protective, and responsible that I’m not just here to get a degree or to get my masters. I’m here to advance our communities and our Knowledges in ways that are responsible, and then, of course, whatever I do, doing it in a way that it matters to our communities, that can be used by our communities. That’s why I’m engaged in a campus and community-based research that’s really connected with our community, our campus and Indigenous leadership. (*Author 3, Sharing Circle*)

5. Self-expression: The value for self-expression and community-driven research means the research conducted in the lab spans many sub-disciplines, and relationships in the IHL build the types of connections necessary to conduct transdisciplinary and wholistic research. “[The lab is] a way of organizing ourselves and building relationships across the sub-disciplines” (*Author 1, Sharing Circle*).

### Allied scholars’ values

6. Compassion and reconciliation:

And so, I think also speaking about the harm of colonized spaces and doing the reflective work to really understand those honestly, and then being a settler scholar I think I have more capacity to look at colonized and neoliberal patterns of relating with a compassionate lens as well because those are absolutely part of me, to be able to find a reconciling middle ground for those. (*Author 2, Sharing Circle*)

7. Personal agency:

And then as far as the values in the lab, they’re challenging for me because it’s new to be in a space that value things do research on our own matters, in our own places, on our own time, and often with our porting one another,” but it’s challenging in a really good way because I know that there are values that are good; good things worth working toward. (*Author 2, Sharing Circle*)

8. Honesty and Humility:

In my professional role I work in land restoration climate change work, and in that role, for four years, I was seeing very extractive ways of relating with Indigenous peoples, who would come to meetings to share and would always come so open[ly]. And that was part of my motivation for becoming an “allied scholar” and learning how to relate in ways that are regenerative and don’t have that extractive pattern. And so, I think as an ally, I’m learning that my role is to continue to be honest with myself about the things in my own way of being and patterns of relating that cause barriers to me being able to be a good ally and noticing when I’m starting to relate in those extractive ways. (*Author 2, Sharing Circle*)

The values identified here serve as common points of connection that guide relationships among Lab members, and in meeting their vision to do research “in a good way.” Achieving this “Vision” calls for new ways of working and enhanced capacity that can be developed through relational and decolonial learning and training. A perceived value-free epistemology and learning environment is incompatible and harmful to Indigenous research sovereignty and capacity building. Interrogation of self and place provides a practice in which allies can critically address the potential power imbalances and epistemic violence that occur in these settings.

To be relational, we *must* first gain consciousness of ourselves and the privilege, energy, and responsibility we have. After all, relationships are not one-sided—and non-Indigenous peoples have created a legacy of mistrust and harm through research “relationships.” As described above, there is a strong foundation of relational and decolonial values that underscore the ways that both Indigenous and allied scholars view their roles and responsibilities both within and beyond the Lab.

#### Learning and practicing decolonizing, geographic health research: Why this matters

In the recent past, the field of geography as a whole, has been responsible for producing research results wherein “a mere 20 years earlier, when individuals and communities were depicted as data points in large surveys” (Richmond and Big-Canoe, 2018: 185). Prompted by a small group of Indigenous and allied scholars in the field, an active movement away from its colonizing project has taken hold, within the emerging sub-discipline of Indigenous geographies, yielding “new cultural geographies [through] a process of re-engagement with issues of Indigeneity through careful, sensitive, inclusive, representative and emancipatory research projects” (Shaw et al., 2006: 267). Within the scope of health geography, is the ability to examine health and relationships from a spatial perspective, seeking to interpret and understand how environments (natural or human built) shape and influence health and well-being.

“Every issue has been approached by indigenous peoples with a view to rewriting and rerighting our positions in history.” (Smith, 2012: p. 29) especially when taking leadership roles in research on matters of direct relevance to their health and well-being. Efforts to respond to this call to action have included establishing deliberate training on Indigenous research sovereignty, globally; prompting scholars to respond, stating “we were urged to do research on our own matters, in our own places, on our own time, and often with our families and communities” (Richmond, 2020: 71).

## Relationship—How we “relate to it”

Indigenous training environments provide essential, emotive and relational spaces of collaborative learning, wherein trainees practice relationship-building, reciprocity, and accountability. When examining the authors’ conceptions of, and experiences with, processes of gathering, learning, and applying lessons to one’s own research projects, the connections between “relationality” and knowledge production become clear. This shift in relational dynamic has been described as “relational accountability” (Reich et al., 2017; Wilson, 2008), and has become a widely accepted ethical imperative and precursor to research, in generating results that help to improve the reality of the research participant (Wilson, 2008: 37).

As discussed in the origins of the IHL, the values and principles carried through the Director, are interwoven and fundamental in understanding how the training environment mirrors the relational framework on which scholars are building their approaches to and within Indigenous communities. As described in Figures 1 and 2, the Vision Wheel, members of the Lab were asked “Do you see the lab as a place of belonging? And if so, what opportunities have come through your relationships, in your research?” As described by the Indigenous scholars, the Lab is an important factor in recruitment and retention of Indigenous health scholars, and the reputation of this space as a research lab matters in fostering community relationship and trust.

I’ve observed that other environments might value “metrics” that are held to a high standard within the Academy, like the number of publications. Whereas I think that we have an opportunity to question whether that’s really valid in terms of saying “that is a measure of success”. I think the things that we’re talking about today are “measures of success,” we’re talking about the opportunity to navigate through tensions when we know we’re coming from a position of limited power within a very hierarchical structure. (Author 1, Sharing Circle)

One way to “measure” whether a space is meeting the needs of scholars is to review feelings of trust or being within a safe and inclusive environment, which we can interpret as descriptions synonymous with the term “belonging.” These are descriptors which are most often referenced, when students’ discuss their “relationships” both in the academy, and more broadly with Indigenous communities.

So, in terms of whether I see this place as the lab as a place of “belonging” and what opportunities have come through those relationships, and how that’s impacted my research, I think being part of a relational environment is part of the learning; so, if we’re learning to do “decolonized research” to move forward in a relational [way] and breakdown hierarchies, in terms of reducing, and minimizing power between the researchers and the communities, I think learning, by being part of a group that values and puts forward “relational approaches” in everything we do is . . . that’s how I’ve have been able to continue to stay in more of a “decolonized” mindset. (Author 1, Sharing Circle)

“Relational” in essence refers to the realization of both context and analysis that will bring about a deeper understanding of the content. “Relational Accountability” refers to processes of how information is surfaced and created in a relational way (through a methodology based within an Indigenous community context), while demonstrating the three “R’s”: Respect, Responsibility, and Reciprocity (to be accountable as it is put into action) (Kirkness and Barnhardt, 1991; Wilson, 2008).

The lab is certainly a place of belonging for me and the research that I engage in; it [is] critical to be a part of a research team that has a strong history of working with [Indigenous] community in ways that have supported . . . [community] interests and desires—their research needs. And so, to be a part of a team that already has that history, that community reputation, has been critical to my research that is examining some “personal experiences” of Indigenous students. So, to come from the Lab and represent the Lab . . . I think has been helpful in people/participants trusting my research. That it’s “not going to sit on a shelf” afterwards; that it’s “a part of community” that’s been created in collaboration with community and . . . the findings will support the community needs [and] campus community needs. So, relationships have been integral in developing the research and executing the research and it’s certainly going to be integral in advancing the study findings and aligning them with the community needs. (Author 3, Sharing Circle)

Senior students (those who have been in the lab for 3 or more years) show a high level of awareness of their role and the value of relationship in supporting community-led research, as well as their own identities and positionality in relation to Indigenous health. That Indigenous training environments provide space for scholars to explore concepts and experiences from a wholistic perspective. The ways relationships are held within the Lab among its members holds the students accountable to be responsible for conducting research with communities in a good way.

Building on my relationship with the [hospital research partner], we are able to create our own understanding of an “Indigenous community” so that we could carry out this research with a group of Indigenous leaders from within the hospital itself, [who] came together to direct me, and they have helped to shape and inform the research goals and objectives, so that it’s not just about what I want to see in terms of research that I think is meaningful within a hospital, because I do come from some place of information, because I’ve had such an extensive career in health care, but I also know that the whole point about Indigenous research sovereignty is really taking the time to listen, and work with the community, in this case with the Indigenous Research Circle within [the hospital] to understand what is going to make a difference for them. (Author 1, Sharing Circle)

Our discussion has been focused on Indigenous health training environments, and the implications that relationality has for supporting Indigenous research sovereignty in our extended web of relations. As identified in the values section, self-reflection and relationality at the intersection of identity are critical components of responsible, decolonial work. This is one example of how this process of reflection can enhance accountability in decolonial work:

Throughout this process I have been reflecting on my future—likely because I face uncertainty on what comes “after” my graduate program. Without a specific destination in mind (i.e., PhD, career, researcher, professional school), my thoughts have circling around positionality, and interrogating colonial spaces—practices that should transcend these academic borders and training environments. I ask myself, “who am I,” and “how do I honour myself and the relationships I have created here as my relations transform?”—I believe are important questions to consider as we move both physically and temporally in our human experience. As a non-Indigenous person especially, it is paramount for me not to replicate or perpetuate colonial harms in relationships. (Author 4, Sharing Circle)

A motivating factor of joining the Lab was seeing and feeling the potential of relationships: across sub-disciplines and sectors, in geographic research, relationship to place, or practitioner settings with relationships to healing spaces, or collaborative relationships between settler and Indigenous peoples.

It’s the fact that I am immersed in this, we’ve created our own “community” within the Lab, so you guys hold me accountable to the work that I do and to the standard of the work that I do. And then also having an Indigenous mentor . . . making space for and time to value the principles, and the practices that are critical in doing community-based research with Indigenous communities and I can reflect on what I see coming from other Labs, in contrast, and I think some of the things that are apparent to me are that there is a different perception on “why” we’re doing this work -the “end goal” is different in some ways. (Author 1, Sharing Circle)

## Knowledge—How we “figure it out”

The IHL is a graduate training environment that supports community-based research and projects that “enable Indigenous communities to address their environment and health concerns” (Indigenous Health Lab, 2022). Research topics include: health and social equity, housing security, environmental dispossession and repossession, food security, Indigenous health geographies, and Indigenous geographies. Currently, the Lab hosts 11 full-time graduate and undergraduate students, including one Indigenous medical student.

### Examples of knowledge—Our research

IHL members shared about their role in community-based research in the “how we ‘figure it out’ context of the Sharing Circle. Specifically, they were asked ‘what has been your experience as a student and trainee?’” We also reflected on the types of knowledge, and specifically of Indigenous knowledge production and methodologies enacted within the lab training environment. In geography, spatial perspectives are fundamental—students’ research projects often reflect their own diverse personal experiences of places, spaces, and systems where Indigenous health and well-being must be improved. One student described coming into their research as a direct result of assisting with the Director’s research, and then pivoting as the impacts of the COVID-19 pandemic started to surface:

My research started off as an extension of my undergraduate thesis on relational accountability and Indigenous health training environments . . . but then the world changed with the pandemic, and so did my research, and we took on this other research project in the summer, going into my Master’s, that interviewed Indigenous physicians and health and social care providers . . . my research now has transformed to “looking at how Indigenous people are connecting across Turtle Island, through social media, to help promote and support mental health during the pandemic-whether it’s through advocacy, resource sharing information, amplifying voices, or just being heard and seen. So, it’s exciting work. It’s definitely new, very inductive, and I’m learning as we go.” (Author 4, Sharing Circle)

My big research problem is addressing an issue that I have experienced and witnessed within Indigenous health care environments. Not because I love hospitals, [but] because they have become places where there has been a lot of harmful treatment mistreatment or lack of treatment towards Indigenous people. [My research addresses how] hospitals are not considered to be safe places, that’s a pretty big paradox considering most people would say that they enter into a hospital for care. (Author 1, Sharing Circle)

### Enacting Indigenous knowledges and worldviews

One allied student describes coming from a western scientific background where her education and professional pathways were often “linear, extractive, reductive and disconnected.” This resonated with other students, who reflected on how, upon encountering Indigenous worldviews, their understandings of the possibilities in terms of how to think about knowledge and approach research were “blown wide open.” To these students, Indigenous Ways of Knowing and Doing seemed more grounded, connected and relevant to “making a difference” in supporting Indigenous sovereignty in research:

My research is taking shape from working professionally in land restoration over four years. A lot of the grants get granted to settler institutions, even though they have explicit deliverables to collaborate with Indigenous communities and Indigenous leaders really are the ones leading good stewardship and what it means to be in good relationship with the land. So [that the funding continues to go to settler institutions] is frustrating to me, [and] that the relationships and collaborations with Indigenous people when they do happen [don’t happen with] a lot of listening or hearing and realigning priorities so that we are adding value to Indigenous health and Indigenous well-being. I recognize [from these experiences] that [in some cases] there wasn’t capacity to have those kinds of conversations and bring in the spiritual, emotional values [and ways of relating to land], yet. (Author 2, Sharing Circle)

Author 1 described how she has experienced coming into a research Lab that values Indigenous worldviews, and how this influenced her research and knowledge practices:

I grew up in a non-Indigenous family. And I’ve had to work really hard to establish relationships and connections back with [Indigenous] community, to feel like I’m “part of.” And I think that that has shaped the way that “I see the world” and “how” and “what I want to know about the world” and I know the Lab has offered me opportunities to draw from “yes,” my Indigeneity—it is valued, my worldview is valued and it’s validated. But I also am learning how to be successful in an academic environment, knowing that it’s only through that combination of the Western processes and really good training in high quality research that we’re [Indigenous scholars] going to be able to get the results that we need, that are going to have an impact on [Indigenous] community. (Author 1, Sharing Circle)

Common threads noted in “Knowledge” identified by students are within the context of relationships with Indigenous mentors, community and peers, recognizing that this network facilitated knowledge transmission, and served as a key support in determining a student’s approach to research. This network of resources extends beyond the Lab, and includes connections with Indigenous mentors from other institutions, communities, and networks. One example is from Author 3, who shares about the process of research project co-creation with community partners:

I received encouragement from my Indigenous mentors at [university] to apply for research funding, I received my first research grant to examine Indigenous student housing needs in London. And from there you know it’s certainly been connecting with the Indigenous campus communities and reaching out for support and giving back. [Then] I made these connections here at [university] and working with Dr. Richmond, we wanted to look at how we can make sure that the research isn’t just “a thesis” that I do and complete on my own . . . how we can connect it with community, how can we make it relevant to our needs here at [university]. We presented the research and I continue to provide updates to the ORGANIZATION NAME and the ORGANIZATION NAME, to make sure that the research is aligned with their needs, and the conversations that they’ve had with students over the many years. (Author 3, Sharing Circle)

From these reflections it is clear students in the Lab conduct research that seeks to build knowledge and capacity, and inform policies which improve Indigenous community health, well-being, and sovereignty. Much of the contemporary research being developed within the Lab is expanding the understanding of the value of relationality and how connections are created within and among community through storytelling methodologies.

The main motivation for my research and the community that [it’s] serving is kind of holding, honoring and being accountable to [the] stories that have been shared with me and finding a way to, builds from the strength [of] these communities and find a way to further support them. (Author 4, Sharing Circle)

## Discussion: Action—How we “do it”

We need to build community amongst peers in training environments to support conducting community-based research. (Richmond, 8 February 2022, Interview by Vanessa Ambtman-Smith, Koral Wysocki, E. Victoria Bomberry, Elana Nightingale, and Veronica Reitmeier [Zoom]).

**Figure 3.**
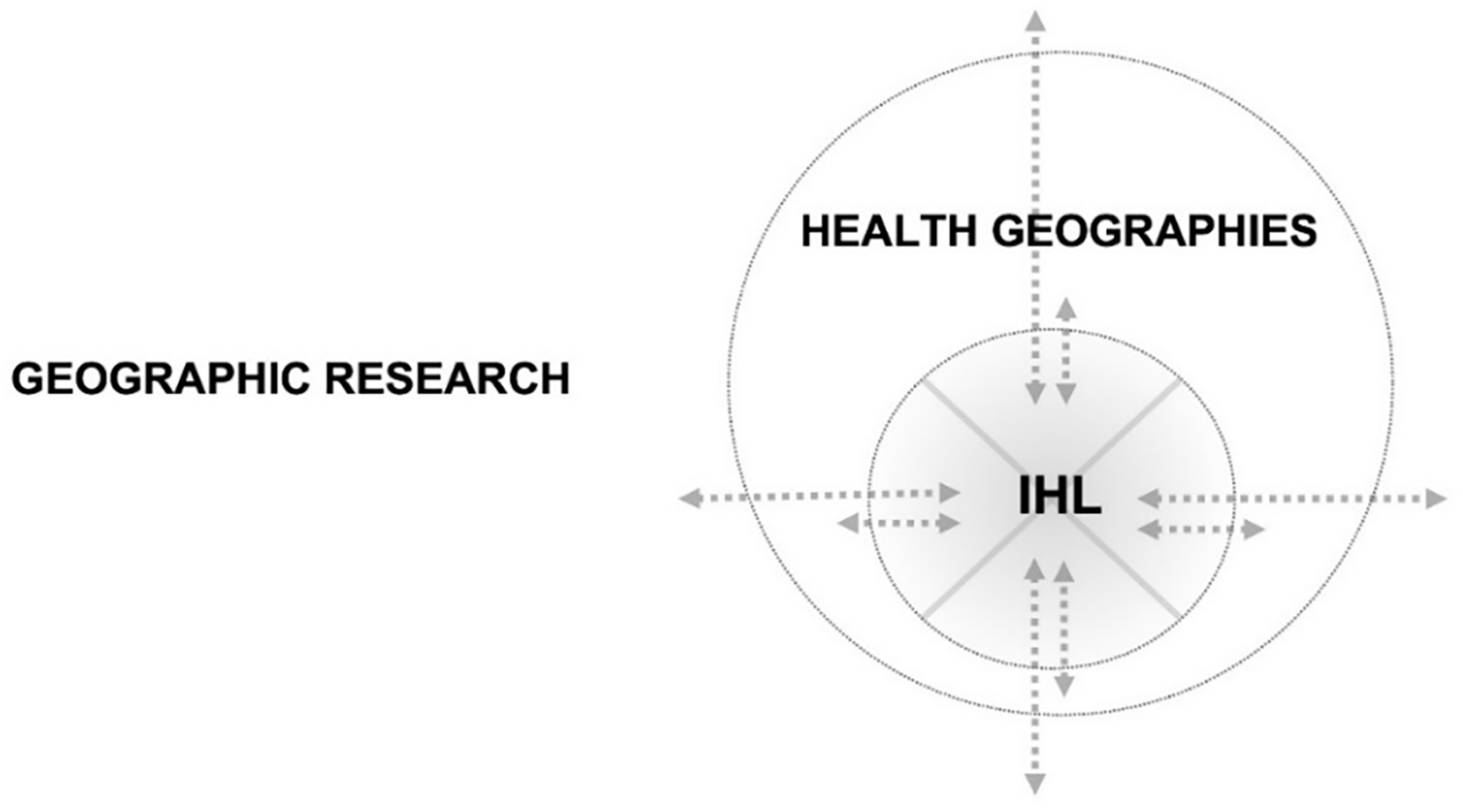
Conceptual positioning of the Lab (IHL) within the broader conceptual fields or “wholes” of Health Geographies and geographic research. The greater and proximal conceptual fields have clearly identifiable influence on the Lab, and vice versa.

**Figure 4.**
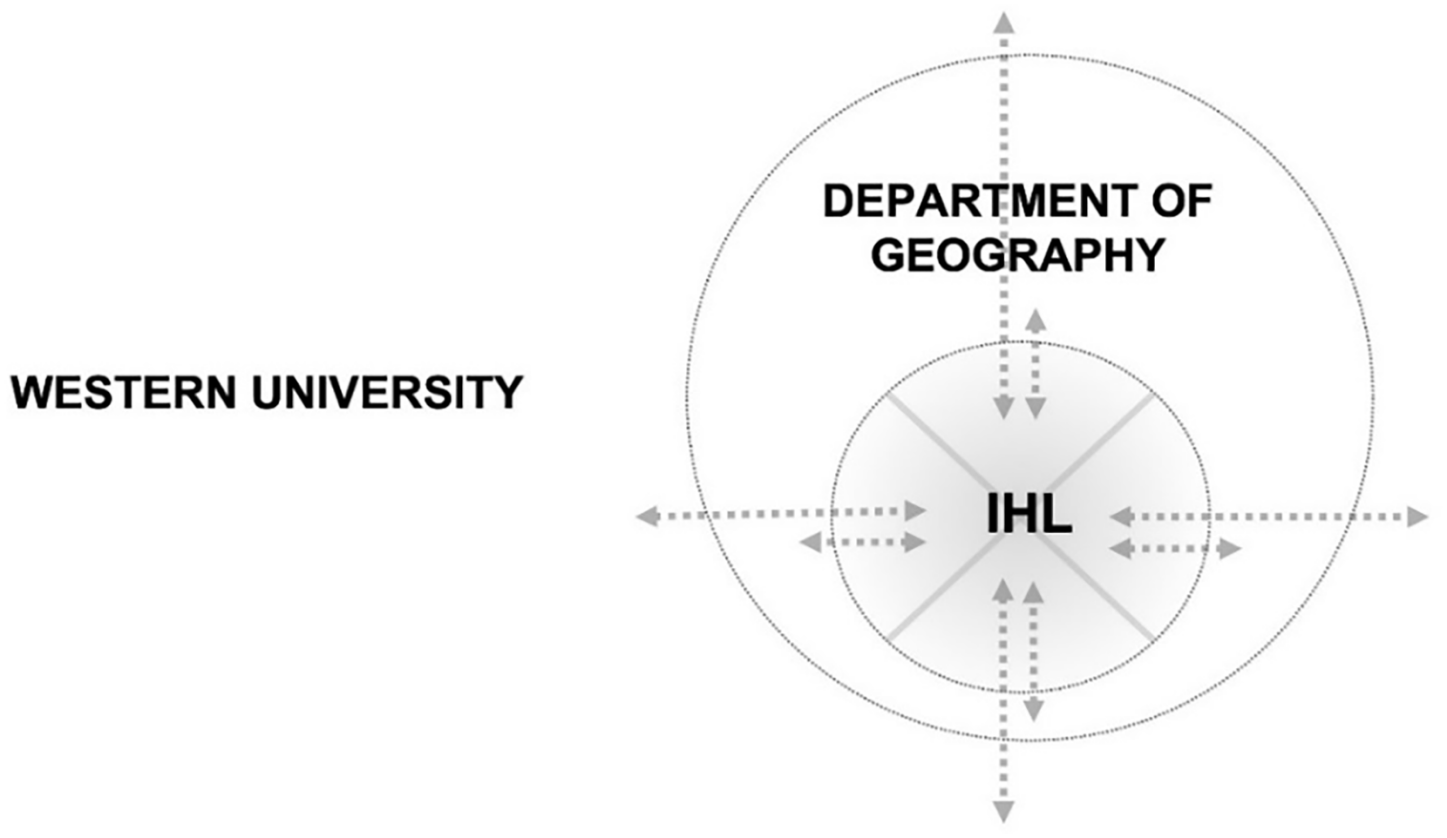
Physical positioning of the LAB (IHL) within the broader physical contexts of the Department of Geography and Environment and Western University. The Department of Geography as an immediate sphere of exchange, with colonial power dynamics limiting the influences of the Lab.

The space and time to do research in a good way with communities allows students the opportunity to develop high-quality research contributions and influence the broader conceptual fields of Health geographies and Geographical research more broadly, as describe below in Figure 3:

Although the Lab is a place and space where students can “think, do and know” in Indigenized ways, it is not a closed system, impervious to the colonial context of the department within which it is situated (as described in Figure 4). Challenges are often surfaced as a result of these contrasting identities and values between the Lab and the department. Outside of the Lab, students experience the erasure and devaluing of Indigenous ways of knowing in classes and administrative spaces. Within the Lab, colonial patterns and insecurities in relationship are surfaced and can show up as resistance to Indigenous worldviews.

At some point conceptually and physically, the identity and values of the Lab come into relationship with the dominant structures, systems, and values of western society. Indigenous students within the Lab noted that have been explicitly encouraged to create healthy boundaries as a method of empowerment to safely navigate harmful tensions and added demands requested of Indigenous students.

And I know that my experience has been enhanced because I have people who can not only help me navigate and talk through options and relate to those tensions, but also just be there for me “emotionally, physically, spiritually, mentally,” and I think that my experience as a student has been very positive . . . I have the capacity to be a “student” here, I have a capacity to be a “learner,” and to know that I am valued as a student and I am going to have the opportunity to learn and grow in that way, without other “expectations” necessarily resting on my shoulders. As an Indigenous scholar, often we get centered out, and we are expected to do things that go above and beyond the role of “student” and I think I’ve had the opportunity to create boundaries that I don’t think would have been as obvious. (Author 1, Sharing Circle)

A common understanding between Lab members is the awareness that relationships are a powerful way to engage an assemblage of people toward realizing a common vision. They are powerful in the sense of the potential for knowledge sharing and the ability for people to help develop each other, in relationship. A lot of our research and work centers relationships among people and between people and place. Relationality of the Lab allows for creative and reciprocally beneficial relationships that bring together scholars as community, and as co-creators, moving them individually and collectively up to a higher expression of their potential.

### Implications within geographic health research and the academy

In making recommendations based on our case study and the information gleaned through sharing, we see three types of implications emerging, which are substantiated through literature, and relevant for both our field of study, as well as colonial institutions: theoretical, methodological, and practical implications. In highlighting these themes, we present a useful synthesis that can be discussed in more environments moving forward.

**Table:** 

Theoretical implications	Quotes	Recommendation
It is through embodied learning, trust, and encouragement that scholars feel that the Lab, as a shared learning environment, cultivates opportunity, brings people in, and advances the type of respectful, and meaningful research critical within the field of Indigenous health geographies. Academic institutions can be bewildering places for many, and for some, can be places of violence, where scholars encounter resistance and racism embedded throughout colonial structures, programs and people.	The Indigenous students describe how in many ways their own research has been inspired through lived experience and shaped through a desire to contribute to meaningful opportunities to redress inequity: “So, my research is bringing forward the Indigenous student housing experiences and needs. I came to the research from my own personal experience in trying to find housing when I wanted to move to the city of Hamilton, to complete my undergrad, and the housing search experience was incredibly demoralizing. I experienced so much discrimination and racism. You know as an Indigenous person you kind of just keep moving because it you know you can’t let it get you too down because there’s going to be more [discrimination and racism] tomorrow” *(Author 3, Sharing Circle).*	The Lab, as a training environment, functions with firsthand understanding of the challenges associated with being “Indigenous in the academy” as well as the rigorous and unique training required to enact decolonial and relationally accountable research practices. It is within these complex and challenging environments that Indigenous and allied scholars often find themselves wholly unprepared to tackle the power differential between students, faculty and institutional structures. Therefore, strong leadership, demonstrating commitment to Indigenous health, well-being and advancing decolonial research through modeling, training, visibility and in supporting students with the appropriate resources needed to do this work, is a critical component for success in navigating through the colonial research environment.
Our deliberate application of an Indigenous framework (Vision Wheel) serves to reinforce why our efforts are both necessary and important. It has been only recently that limitations within the scope of the natural and social sciences have been documented from an Indigenous perspective, where it is noted that researchers have historically been outsiders who seek to “study” Indigenous problems using the widely supported scientific approach (Smith, 1999). Through our choices in collecting reflections and presenting back information in this way, we have created a paper that models our learnings, centering an Indigenous epistemology and methodology (Wilson, 2001), and thereby creating a narrative that we believe will resonate with Indigenous and allied scholars alike.	What has been recognized is that with this “Western, outsider approach”, the researcher themselves, coupled with the methodology(s) employed will carry biases, whether implicit or explicit, shaping the type of knowledge produced, and offering a “Eurocentric definition of reality upon the rest of the world” (Wilson, 2008: 16). It is through this version of reality that we have seen the growth and “proliferation of negative stereotypes about Indigenous communities” (Wilson, 2008: 17).	The Lab was created as a training environment to support Indigenous health research. The Lab draws in graduate scholars looking to engage their whole selves in research that supports communities, research that requires scholars to learn with their heart and mind (Richmond, 2020). Although it is notably challenging for students to undertake research in this way with relatively short degree timelines (2–4 years), a commitment to research grounded in a space and place that honors whole, self-expression, cultivates belonging and allows a relational approach to knowledge co-creation, the Lab is guided by values that strengthen the relationships we work hard to cultivate. Research sovereignty and community governance require space and time to develop.
When reflecting on the growth of their own perspectives and capacity to engage in relational approaches, there was a depth and contrast in what the authors’ shared about their experiences within and outside the Lab. Indigenous students identified that their encounters within academia and professional workplaces were often marginalizing at best, and at worst, violent, harmful and isolating. Often, “identity” was reduced to “one thing” or only provided space and value for “one narrative.” The authors recounted feelings of not belonging, and a desire to flee academia prior to finding their place of belonging within academia through the Lab.	“As a training environment as a ‘place’ where I can say, ‘I’m part of this group’, we have a valid and legitimate connection in the Academy, that’s respected. We have that track record that [other student] speaks of and people know ‘yeah, okay we trust you, we know that this is going to be good work’ because of Chantelle’s leadership and her training; she’s not going to let me just get away with work that’s not a high quality, and I think that it’s a reflection on her commitment to community, that our group is cultivating a high quality level of research and excellence that is not only going to be important in informing our department here but also we’re putting it out back out into the world and showing others that they can do the same” *(Author 1, Sharing Circle).*	The Lab runs in contrast to the status quo, and thus, having the support of leadership, space and time to do research in a good way with communities is imperative in redressing power dynamics that could hinder such efforts otherwise, allowing students the opportunity to develop high quality research contributions and influence the broader conceptual fields of Health geographies and geographical research more broadly, through this safe working space.

### How we navigate through colonial tensions

The Lab is an active place of learning, which includes learning how to thrive within a colonial environment, where connections extend through individual identities, living experiences, and within an institution that privileges the colonial project.

[After an] offensive experience [in a graduate-level class], that [made me] feel like I [didn’t belong] in graduate school or at UNIVERSITY, the support I received from NAME and the team made me see that I have valuable contributions even if course instructors didn’t realise it and that part of what my role is here as an Indigenous scholar is to disrupt conventional ways of seeing and thinking about the world that have intentionally erased Indigenous knowledge and worldviews. I certainly wouldn’t have felt as welcomed or as valuable as an Indigenous scholar on campus if it weren’t for the lab. (Author 3, Sharing Circle)

Elements of racism, sexism, eurocentrism, colonialism, and neo-liberalism continue to dominate, manifesting real and violent tensions that can impact and derail efforts that unsettle these patterns. There is power in community. Creating a place where Indigenous voices are valued, and scholars are encouraged to “come in and think about the big questions, and be helpful,” (Richmond, 2020) is fundamental to creating a safe space for learning and forming relationships across the Lab. In this way, the Lab can be described as an extension of this relational philosophy, wherein people are welcomed in, encouraged to identify and use their natural gifts in shaping and forming their own unique and courageous paths forward.

One of the fundamental [realizations] that has shaped [me] as an ally, [is the need to] constantly be aware [and reflexive] with your perspectives [to realize how] they [shift and] transform over time. And a lot of [those shifts in perspective come] from relationships and teachings and [those are] things that you carry with you. And so, I guess this comes back to what I said about “learning with your heart.” (Author 4, Sharing Circle)

The work of negotiating and cultivating a respectful research relationship is iterative. There is no one way to go about enacting relational accountabilities. Therefore, appropriate mentorship, training, and listening must be part of the learning process. “Having good intentions about my research relationships is not sufficient. Even in instances when researchers have every intention of honouring and valuing Indigenous collaborators, good intentions do not always lead to respectful actions” (Reo, 2019: 5). As exemplified in the following quote, an Indigenous scholar notes:

The lab has helped me to know and understand that who I am and what I do matters, and that it is no coincidence that I am able to bring my “whole-self” into this environment and into this research because, as Shawn Wilson shares with us “we are the relationships in research”; these things are not disconnected and it really validated the way that I see myself in the world. (Author 1, Sharing Circle)

It is through this collective model that scholars are trained to engage in Indigenous Ways of Knowing and Doing, and the application of decolonizing approaches to research and practice, building competency and capacity to shift mindsets and attitudes around the value of relational accountabilities in geographic research (Castleden et al., 2017). This knowledge framework includes “placing Indigenous peoples at the centre of the research environment and is cognizant of Indigenous values, beliefs, paradigms, social practices, ethical protocols, and pedagogies” (Denzin et al., 2008: 92).

## Conclusion

We end this article by sharing about our mentor and their experience establishing the Lab. The establishment of the IHL, as a space that attracts and nurtures Indigenous and allied scholars, did not happen by chance: the Lab came to be through the difficult negotiations, leveraging external awards and grants to build a undeniable rationale for why a space for Indigenous and allied scholars engaged in Indigenous research was needed. In fact, this institution was selected specifically “because the things that I care about, and the mission that I’m on does not exist [here]” (Richmond, *8 February 2022, Interview by Vanessa Ambtman-Smith, Koral Wysocki, E. Victoria Bomberry, Elana Nightingale, and Veronica Reitmeier [Zoom]*). This mission, to provide a welcoming space where students can embrace who they are and feel good about where they come from, centering Indigenous Ways of Knowing led to an environment created to prepare emerging scholars and trainees in how to practice relationality in research. We believe the Lab has created a culturally-safe space and “unity of environment” to address the “trickiness” of settler-colonial tensions and relationships, navigating and confronting barriers to relationality that are important skillsets in supporting Indigenous self-determination and research sovereignty beyond the academy. Indigenized and decolonizing spaces have the potential to nurture many dimensions of growth and identity, culturally, emotionally, spiritually, and mentally, wherein scholars describe “be[ing] valued as a researcher and as a person,” connecting their “whole selves” into academic training that is designed to redress and transform former harmful research practices. As described by Dr. Richmond, there is a mission and purpose for the Lab that is relational:

At the heart of this research training environment is something different, something more human, guided by dimensions, principles and values that mirror the ones that guide relationships in life, because you don’t become a different person because you enter a different environment . . . you can’t just bring people here and then not feed them. (Richmond, 8 February 2022, Interview by Vanessa Ambtman-Smith, Koral Wysocki, E. Victoria Bomberry, Elana Nightingale, and Veronica Reitmeier [Zoom])

We have now embarked upon a “new culture of Indigenous research,” facilitated through intentional Indigenous research infrastructure. This movement has been sparked by the development and dissemination of Indigenous health training resources by national leaders such as the Canadian Institutes of Health Research. Through leaders like Dr. Richmond, Indigenous scholars have created, or are seeking to create, opportunities to build on an Indigenous community health agenda which “privileges the voices at the ground-level,” employing both Indigenous and decolonizing methodologies. These are emergent practices, with the first generation of Indigenous health network funding emerging in the mid-2000s. We recommend further study to understand how these training environments (at the national, regional, and institutional levels) can provide opportunity for Indigenous and allied students to gather, learn, grow, and build a community of practice that celebrates and prioritizes Indigenous contributions to research. These research philosophies and practices have been widely adopted as a minimum standard for ethical research with Indigenous communities, and are requirements underscoring most institutional ethics reviews, and tri-council funding agencies in Canada (Canadian Institutes of Health Research et al., 2018). There is a deficit of literature and learnings based on the student-perspective, which we know is foundational in understanding what these training spaces look like, how they are used, and what they mean for Indigenous and allied health scholars embarking on their academic journeies.

It is no accident that many of the sentiments expressed by current scholars in this publication reflect the transformative potential related to larger networks of Indigenous scholarship (e.g. ACADRE, NEAHR, and IMN), knowing that these models themselves influenced the Lab’s Director in her own pathway forward from Indigenous trainee to an independent Indigenous health scholar (Richmond, 8 February 2022, Interview by Vanessa Ambtman-Smith, Koral Wysocki, E. Victoria Bomberry, Elana Nightingale, and Veronica Reitmeier [Zoom], p. 5). Drawing on results presented through ACADRE and NEAHR participant experiences, there is good evidence to support the value and benefit of programs that create “the time, space and resources [students] need to learn, see and personally experience research through Indigenous ways of knowing” (Richmond et al., 2013.)

As we are near the end of the third generation of Indigenous health network funding, we view our Lab as a generative environment that is connected to the work and legacy of these larger training networks. It is through these networks that we may access a larger knowledge base on student and trainee experiences by examining the platforms that create capacity to engage in hopeful and helpful research.
